# Optimizing Treatment for Adults with HIV/AIDS in China: Successes over Two Decades and Remaining Challenges

**DOI:** 10.1007/s11904-019-00478-x

**Published:** 2020-01-14

**Authors:** Wei Cao, Evelyn Hsieh, Taisheng Li

**Affiliations:** 1 Department of Infectious Diseases, Peking Union Medical College Hospital, Chinese Academy of Medical Sciences, Beijing, China; 2 Section of Rheumatology, Department of Internal Medicine, Allergy and Immunology, Yale School of Medicine, New Haven, CT USA

**Keywords:** Human immunodeficiency virus, Acquired immune deficiency syndrome, Highly active antiretroviral therapy, National Free Antiretroviral Treatment Program, Multidisciplinary care, Continuum of care

## Abstract

**Purpose of Review:**

The introduction of the National Free Antiretroviral Therapy Program (NFATP) in 2003 by the China National Center for AIDS/STD Control and Prevention has led to dramatic increases in antiretroviral therapy (ART) coverage among HIV-infected Chinese patients. Despite limitations in the number of available free antiretroviral drugs, the overall mortality associated with HIV/AIDS has dropped from 39.3 per 100 person-years in 2002 to 3.1 in 2014. In this review, we summarize the challenges, responses, and achievements of antiretroviral therapy (ART) in China over the past 20 years.

**Recent Findings:**

Continuous optimization of the Chinese National Guidelines for HIV/AIDS Diagnosis and Treatment has been guided by data from serial domestic multi-center studies aimed at evaluating efficacy and toxicity of available ART regimens among Chinese patients with HIV, with the goal of maximizing adherence, access, and efficacy. In addition, increasing attention has been focused on the importance of continuity in the HIV care cascade to promote linkage to care, and address the multidisciplinary chronic care needs HIV/AIDS patients on lifelong ART.

**Summary:**

Great progress has been achieved in the past 20 years in terms of access to and optimization of antiretroviral treatment in China. As the number of patients receiving long-term ART continues to grow, the focus of HIV/AIDS treatment has gradually transitioned from urgent care to the management of non-AIDS-related chronic complications and control of chronic inflammation.

## Introduction

In 1985, the first case of acquired immunodeficiency syndrome (AIDS) in China was reported by Peking Union Medical College Hospital in Beijing [1]. Since then, the Chinese HIV/AIDS epidemic has seen a rapid increase, especially in the last decade. The pattern of the Chinese HIV epidemic has shifted over the past 35 years. In the first decade, HIV outbreaks were concentrated among the contaminated blood product donors/recipients. However, since the late 1990s, sexual contact has become the predominant driver of transmission, initially mainly via heterosexual transmission. More recently homosexual transmission among men who have sex with men (MSM) has played an increasingly significant role. By the end of 2018, there were an estimated 1,250,000 people living with HIV in China, with 80,000 new infections reported in 2018, an increase from 57,194 new cases in 2017 [2]. The continued rise in HIV incidence highlights the ongoing challenges of HIV/AIDS control in China.

Although the groundbreaking strategy of highly active antiretroviral therapy (HAART) was identified as early as 1996, standardized HAART was not available to the vast majority of Chinese patients until 2003 when the national AIDS control policy “Four Frees and One Care” was announced and the National Free Antiretroviral Treatment Program (NFATP) established [3]. The NFATP rolled out its pilot programs in six provinces of Central China and targeted former blood and/or plasma donors, but quickly scaled up to cover patients with HIV across the country [3]. By late 2006, the NFATP encompassed all 31 provinces and autonomous regions of China, and 64% (30,640/47,713) of all patients diagnosed with HIV/AIDS had been initiated on free government-sponsored ART [4]. With rapid expansion and continual evaluation and adjustment of the program, China has successfully reduced the overall mortality of patients with HIV/AIDS from 39.3 per 100 person-years in 2002 to 14.2 in 2009, despite a limited repertoire of available antiretroviral drugs. Nevertheless, numerous operational problems and resource limitations have been encountered during implementation of this program. In response, a series of government-sponsored, multi-center studies have been carried out over the years to guide optimization of the HIV/AIDS treatment approach for Chinese patients, with the aim of combating China’s HIV/AIDS epidemic and achieving the last of the World Health Organization’s (WHO) ambitious 90-90-90 targets.

### Optimizing HAART for Chinese Patients with HIV

Prior to the nationwide launch of the NFATP in 2003, experts first had to address the critical question of which antiretroviral regimens to select for Chinese patients. Initially, the only available antiretroviral drugs in China were domestically produced generic zidovudine (AZT), stavudine (d4T), didanosine (ddI), and nevirapine (NVP). Therefore, the original NFATP first-line regimen, given to approximately 80% of HIV-infected patients, consisted of AZT + ddI + NVP. The other 20% received d4T + ddI + NVP, primarily in the setting of hematologic contraindications or adverse reactions to AZT. Domestically produced, generic indinavir (IDV) and imported, brand-name efavirenz (EFV) and lamivudine (3TC) were added subsequently to the list of NFATP-sponsored free antiretrovirals. However, there was no evidence with regard to the optimal treatment regimen for Chinese patients due to the paucity of data regarding safety and efficacy of these drugs in this population.

In 2004, the China HIV/AIDS Clinical Trial Network (CACT) was created, initially involving 17 hospitals and health agencies nationwide, including both local referral centers and those covering areas with high HIV prevalence. With support from the Ministry of Science and Technology’s National Key Technologies R&D Program over the past 15 years (via the 10th–12th Five-Year Plan grants), the CACT has carried out a series of multi-center clinical trials and cohort studies among HIV-infected adults over China. Between 2004 and 2006, the first multicenter prospective controlled HIV/AIDS trial was completed in China, in which 198 treatment-naïve Chinese patients with HIV/AIDS were recruited and randomized to three HAART regimens composed of three of the four aforementioned available antiretrovirals and followed for at least 1 year. Results showed that d4T + 3TC + NVP and AZT + 3TC + NVP achieved a viral suppression rate of approximately 70%, significantly higher than that of d4T + AZT + NVP. Furthermore, these combinations only cost one sixth of the price of imported drugs [5]. As a result, d4T + 3TC + NVP and AZT + 3TC + NVP became the initial recommended first-line regimens in China, and were incorporated in the first Chinese National Guidelines for HIV/AIDS Diagnosis and Treatment published by the Society of Infectious Diseases, Chinese Medical Association.

In the first few years following implementation of NFATP, progress was limited by a high incidence of adverse effects and poor adherence. Severe hepatoxicity largely attributed to NVP presented as the most common short-term adverse effect leading to ART interruption. The WHO recommended avoidance of NVP in women with CD4 cell counts ≥ 250 and men ≥ 400 cells/mm^3^, to reduce the incidence of serious NVP-related adverse reactions of the skin and liver. However, CACT data demonstrated that NVP-associated hepatoxicity in Chinese patients showed little difference between sexes, and increased substantially when the pre-treatment CD4 cell count exceeded 250 cells/mm^3^ in both women and men [6]. According to these observations, the 2011 Chinese National Guidelines for HIV/AIDS Diagnosis and Treatment recommended that providers weigh the benefits of NVP carefully against the risk of severe hepatoxicity in patients with baseline CD4 counts above 250 cells/mm^3^ regardless of the patient’s sex [7]. Following this recommendation, drug-related hepatoxicity was reduced by 50%, which in turn markedly improved patient adherence.

In the setting of extremely limited antiretroviral options, treatment adherence was recognized as the cornerstone of achieving sustained virologic control. In addition to patient education and adherence monitoring, strategies to minimize drug-related adverse effects played a critical role in promoting adherence. As experience accumulated from patients enrolled in the NFATP, it was observed that lipodystrophy—the major side effect of d4T—usually manifested after 6 months of treatment, whereas bone marrow suppression from AZT exposure occurred soon after ART initiation, partially due to the fact that most Chinese HIV/AIDS patients were diagnosed at a relatively late stage and initiated on treatment when they were more susceptible to bone marrow suppression. Therefore, CACT researchers proposed a “d4T to AZT switch regimen” consisting of six months of d4T + 3TC + NVP followed by AZT + 3TC + NVP for long-term maintenance, to take advantage of the time gap between the two adverse effects and minimize the impact of each one. In an open-label study, 517 treatment-naïve HIV-infected patients were recruited and randomized to receive either AZT + 3TC + NVP, d4T + 3TC + NVP or the aforementioned “switch regimen.” At the end of 96 weeks, the switch regimen showed a comparable level of viral suppression and CD4 recovery, with 80% reduction in bone marrow suppression compared with the AZT-based regimen, and over 90% reduction in lipodystrophy compared with the d4T-based regimen [8]. This trial demonstrated that using the same repertoire of drugs but adjusting the order of administration led to a feasible, effective, and affordable therapeutic option especially in resource-limited settings. This regimen was subsequently also incorporated into the 2011 national guidelines as alternative regimen, and provided to over 27,000 Chinese treatment-naïve patients in ensuing years with significantly decreased toxicity and greatly improved adherence [7].

During the same period, in the setting of WHO recommendations for tenofovir-based ART regimens for resource-limited settings, combination TDF + 3TC + EFV was newly adopted as first-line therapy in the 2011 Chinese National Guidelines for HIV/AIDS Diagnosis and Treatment and was concurrently made available through the NFATP. The efficacy and tolerability of this regimen for first-line therapy in Chinese patients was studied and confirmed in a multi-center cohort study carried by the CACT (Clinicaltrials.gov ID: NCT01844297). Although EFV was approved by the China Food and Drug Administration (CFDA) as early as 2005, usage was low prior to 2011 due to cost. As EFV use increased after inclusion in the NFATP, a growing number of patients reported moderate to severe neuropsychiatric symptoms with the standard 600 mg daily dose of EFV, including dizziness, insomnia, nightmares, and depression, as has been reported internationally. The multi-national ENCORE1 studies published in 2014 and 2015 first suggested the non-inferiority of EFV 400 mg to EFV 600 mg daily [9, 10], and in a prospective study by the CACT, plasma EFV levels measured in 455 treatment-naïve HIV patients initiated on an EFV-containing regimen showed that 34.2% and 43.8% of participants had plasma EFV concentrations above the upper limit of the proposed therapeutic window at 24 and 48 weeks, respectively, especially in those with low body weight (< 60 kg) [11]. Based on these collective findings, the 2018 Chinese National Guidelines for the Diagnosis and Treatment of HIV/AIDS suggested a dose adjustment for individuals with low body weight [12], and a prospective open-label randomized CACT trial of EFV 400 mg versus 600 mg aimed at collecting real-world evidence among Chinese patients with HIV has just completed enrollment.

The NFATP-sponsored regimens have undergone continuous evaluation and improvement over the past 16 years, and greatly expanded access to treatment and therapeutic options (Table 1). Data from domestic studies such as those of the CACT network have provided important guidance. This, in parallel with efforts from the Chinese Society of Infectious Diseases to develop and update the national guidelines for HIV/AIDS diagnosis and treatment, and to carry out national training campaigns for healthcare providers, has greatly improved the capacity and quality of HIV/AIDS care in China (Table 2). In recent years, CFDA approval of new categories of ART drugs including the integrase inhibitors, entry inhibitors, and new generations of protease inhibitors have provided more options especially for those faced with drug-related adverse effects or drug-drug interactions. The indication for ART initiation has also changed with time. When the NFATP was first established, the indication for ART initiation was a CD4^+^ T cell count less than 200 cells/mm^3^ or advanced AIDS status. The CD4^+^ T cell count threshold for ART initiation was increased to 350 cells/mm^3^ in 2011, 500 cells/mm^3^ in 2015, and since 2016, China has entered the era of immediate treatment irrespective of CD4^+^ T cell count, in accordance with most international guidelines. In addition to the international findings from the START study, local epidemiological findings further justify early ART initiation in China [15]. Over the past 20 years, there has been a notable shift in the distribution of HIV genotypes in China. The proportion of patients with circulating recombinant strain CRF01_AE has increased significantly, and since 2006 has become the most prevalent genotype, especially among patients who acquire HIV through sexual transmission [16]. The CRF01_AE subtype is associated with a more rapid decline in CD4^+^ T cell counts and faster disease progression, with a median of 4.8 years from estimated date of infection to onset of AIDS, in contrast to the previously predominant C or BC subtypes, which have a well-established latent period of 8–10 years [17]. This biological characteristic of the epidemic in China further underscores the importance of strategies to promote timely HIV testing and early treatment initiation.

**Table 1:** Current ART drug availability in China

Drug class	Available in China		Not available in China
	Free	Not free	
NRTIs	Lamivudine (3TC) Tenofovir (TDF) Zidovudine (AZT) Abacavir (ABC)	Emtricitabine (FTC) Tenofovir Alafenamide (TAF)	
NNRTIs	Efavirenz (EFV) Nevirapine (NVP)	Rilpivirine (RPV) Etravirine (ETV)	Doravirine (DOR)
PIs	Lopinavir/ritonavir (LPV/r)	Darunavir/cobicistat (DRV/c) Atazanavir (ATV)	Fosamprenavir (FPV) Nelfinavir (NFV) Saquinavir (SQV) Tipranavir (Aptivus)
INSTIs		Raltegravir (RAL) Dolutegravir (DTG)	Bictegravir (BIC) Elvitegravir (EVG)
Fusion inhibitor		Albuvirtide	Enfuvirtide (T20)
CCR5 antagonist			Maraviroc (MVC)
Co-formulated fixed-dose	AZT/3TC	FTC/TDF FTC/TAF AZT/3TC/ABC ABC/3TC/DTG TAF/FTC/EVG/c	BIC/TAF/FTC EVG/c/TDF/FTC DTG/3TC DRV/c/TAF/FTC EFV/TDF/FTC RPV/TDF/FTC

**Table 2:** Recommended first-line and alternative ART regimens in China’s national guidelines for HIV/AIDS treatment (2005–2018)

Guidelines	2005 [13]	2011 [7]	2015 [14]	**2018[**12**]**
First-line	AZT(d4T) + 3TC+ EFV(NVP)	TDF + 3TC + EFV +LPV/r +RAV or ETV	TDF(ABC) + 3TC (FTC) + EFV +LPV/r or ATV +RAL	TDF(ABC) + 3TC (FTC) + EFV or RPV TAF + FTC + LPV/r or DRV/c +DTG, RAL Co-formulated: TAF/FTC/EVG/c ABC/3TC/DTG
Alternatives	AZT(d4T) + 3TC + IDV ddI + d4T + EFV(NVP) AZT + ddI + EFV(NVP)	AZT + 3TC + NVP d4T + 3TC + NVP (Switch to AZT/ABC +3TC after 6 months)	AZT + 3TC + EFV or NVP or RPV	AZT + 3TC + EFV or NVP or RPV +LPV/r

### Promoting Multidisciplinary Care and Integrating the HIV/AIDS Care Cascade

Despite continued challenges with achieving early diagnosis of HIV among infected individuals in China, once diagnosed, improved access to ART has meant that HIV/AIDS is no longer a fatal condition for many patients, but a chronic disease that can be well managed with long-term treatment. As a result, the life expectancy of HIV-infected individuals has extended dramatically in China as it has in other regions of the world. However, increased survival is also associated with a higher incidence of non-AIDS-related morbidity and mortality which is not directly attributable to immunosuppression. A meta-analysis by Teeraananchai *et.al.* found substantial improvements in life expectancy among individuals with HIV in the ART era, particularly in high-income countries, although life expectancy was not quite that of the general population across all regions [18]. Non-AIDS-related comorbidities including cardiovascular, renal, respiratory, bone and neurological diseases, metabolic syndrome, non-AIDS-defining malignances are becoming a new focus of HIV/AIDS care that directly impacts patients’ quality of life.

In this context, beyond ART, comprehensive multi-system screening and management has been recognized as an increasingly important component of HIV care. On the one hand, HIV infection and its associated immune activation has been identified as an independent risk factor for many non-AIDS complications, including atherosclerosis, cardiovascular diseases, osteoporosis, and renal diseases [19–24]. On the other hand, lifelong use of ART also exerts systemic influences including but not limited to alterations in lipid and glucose metabolism, liver and renal functions, bone and mineral homeostasis, and neuropsychiatric changes. Moreover, individuals will inevitably face significant challenges with polypharmacy and drug-drug interactions as they manage the dual burden of HIV infection and concurrent non-AIDS-related comorbidities. Therefore, multidisciplinary management has been recognized as the future direction of HIV/AIDS care in China, and a few major HIV care centers have already started piloting strategies to achieve this. During the past few years, medical specialty societies have also started working together, and expert consensus papers and recommendations regarding screening and treatment of renal complications in HIV and mycobacteria coinfection have already been published [25–27]. Additional collaborative recommendations focusing on cardiovascular complications, osteoporosis and fracture, hepatitis C coinfection, and others are also in progress. These collaborative initiatives serve a dual purpose. First, they provide important knowledge and guidance for HIV care professionals regarding diagnosis and management of non-AIDS-related comorbidities. Second, and perhaps more importantly, they have engaged and educated health professionals from diverse medical fields in HIV care. The latter represents an important means of breaking down the stigma that many patients in China face when seeking care outside of their HIV care centers.

Due to the endemic nature of hepatitis B in the Asia-Pacific region, HIV- and HBV-coinfected patients have historically been an important subpopulation in China. However, when ART was first introduced in China over 20 years ago, there was very little knowledge about the epidemiology, natural history, and management needs of this group. The epidemiological map of HIV/HBV coinfection was clarified through serial studies. According to two national multicenter studies that enrolled a total of 35,849 adult patients with HIV, 8.7–9.4% were coinfected with HBV, with much higher prevalence in the eastern and southern regions of China [28, 29]. Coinfected patients demonstrated more significant immunosuppression and more rapid HIV progression compared with HIV mono-infected individuals. Fortunately, virological and immunological responses to ART were not affected by HBV coinfection status among Chinese patients [30]. In terms of treatment, prior to widespread availability of TDF through NFATP, the efficacy of using a regimen that included only one active agent (3TC) for HBV coverage was demonstrated among Chinese patients in a study by Li and colleagues, and 3TC alone could be considered for HBV coverage in HIV/HBV coinfected patients when baseline HBV DNA levels are lower than 20,000 IU/mL, providing a feasible treatment option in resource-limited settings [31]. The current recommendation is to select a HAART regimen that includes two drugs with anti-HBV activity.

**Fig. 1:**
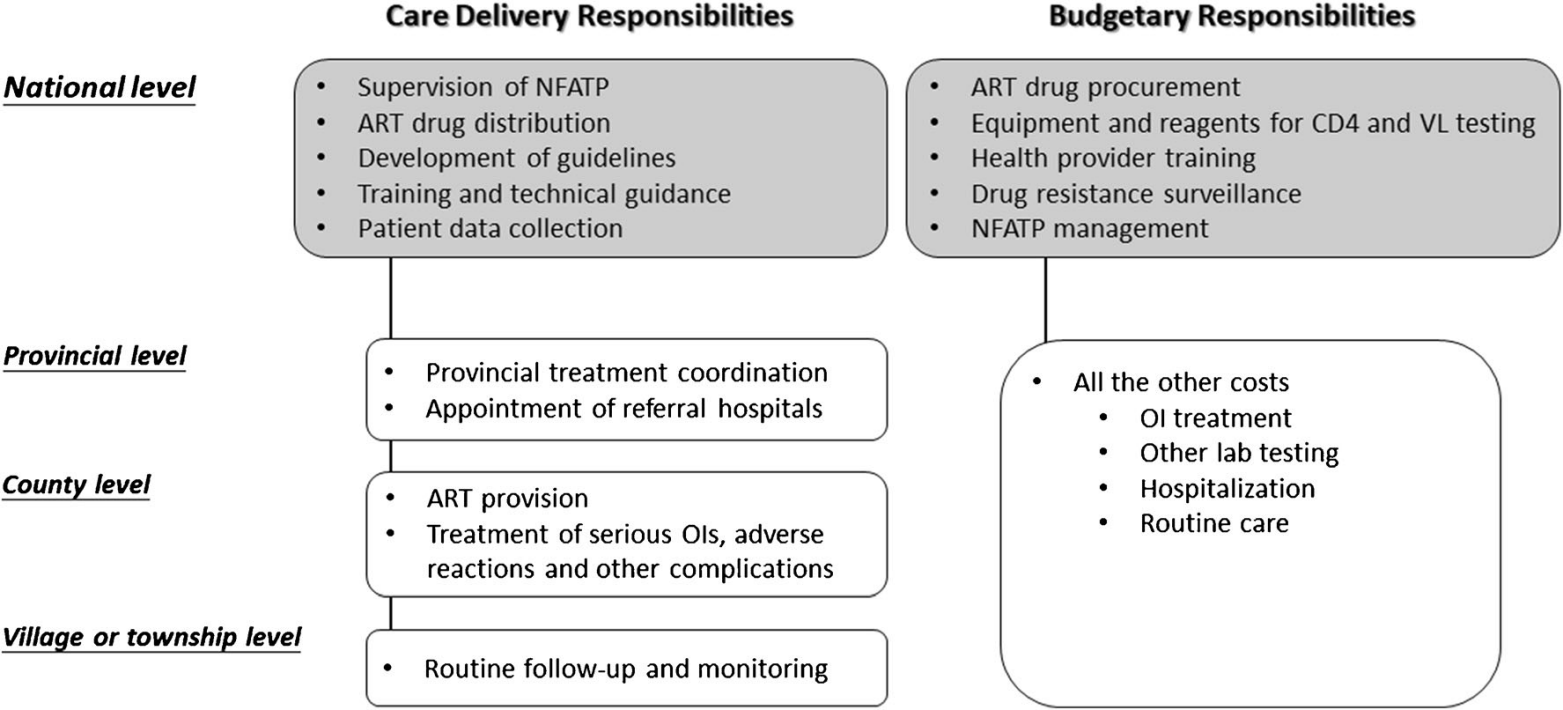
Structure of care delivery and budgetary responsibilities at different administrative levels of the NFATP. Adapted from [4]

**Fig. 2:**
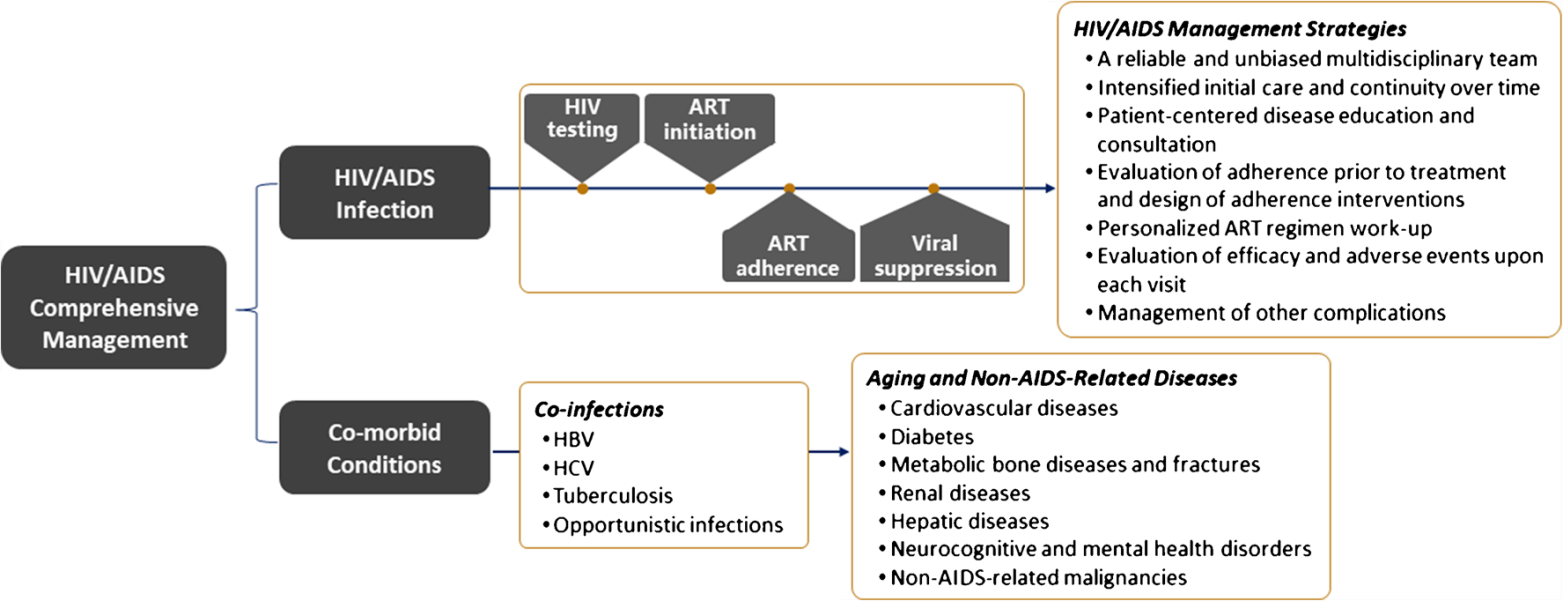
Proposed model for comprehensive management of HIV/AIDS in China

Traditionally, HIV/AIDS surveillance and diagnosis services have primarily been the purview of China Centers for Disease Control and Prevention (CDC) units, whereas treatment and follow-up care occur at designated health care facilities (Fig. 1). However, this has created a physical separation between the sites where patients are diagnosed and the sites where they receive treatment and long-term management, posing a structural barrier to linkage of care. In the 2018 Chinese National Guidelines for HIV/AIDS Diagnosis and Treatment, the concept of an integrated care cascade was first introduced, encouraging coupling of diagnosis with treatment and management services within HIV care centers, and engagement of a coordinated, multidisciplinary team to ensure the provision of comprehensive care (Fig. 2) [12]. The envisioned, patient-centered model would minimize redundancy, emphasize continuity of care and promote adherence, all ultimately leading to improved HIV control and outcomes. Key components would include prevention and prompt diagnosis of HIV infection; diagnosis, and treatment of opportunistic infections and AIDS-defining malignances; initiation of personalized ART, adherence monitoring and follow-up; screening and management of non-AIDS-related diseases; and social and psychiatric supports and interventions (Fig. 2).

### Remaining Challenges and Future Directions

China has made great achievements in the fight against HIV/AIDS in the last two decades. According to the progress report from National Center for AIDS/STD Control and Prevention of the China CDC, by the end of 2017, a total of 610,000 HIV-infected individuals were receiving ART in China, accounting for 80.4% of all the reported cases, and over 90% of those on ART achieved successful virologic control [2]. The mortality of HIV/AIDS patients in China has decreased from 22.6 per hundred-person-year in 2003 to 3.1 per 100 person-year in 2014. Meanwhile, the increasing availability of domestic data from research networks such as the CACT and others have directly informed the development of treatment guidelines that take into account the specific biology of Chinese patients with HIV and the realities of the health care environment in China. The 2018 Chinese National Guidelines for HIV/AIDS Diagnosis and Treatment has been the third update of the guidelines within 10 years. However, despite the tremendous progress achieved, potential problems and risks still remain.

#### Late Presenters and Delayed Diagnosis

Although regular surveillance of key populations is an important part of the national approach to case finding, delayed diagnosis of HIV/AIDS remains an important issue in many regions, particularly in areas where healthcare services are limited. The clinical latent period from HIV acquisition to the onset of AIDS, and the non-specific kaleidoscope of symptoms that patients may initially present with, make early diagnosis extremely challenging in the absence of routine screening programs. Although awareness of HIV infection among healthcare providers has been greatly improved in general, a large proportion of infected individuals still have very low CD4^+^ T cell count at diagnosis suggesting missed opportunities for timely diagnosis. According to data from Chinese Comprehensive Response Information Management System of HIV/AIDS (CRIMS), 34.0% of newly diagnosed patients with HIV/AIDS between 2006 and 2014 presented with CD4^+^ T cell count below 200 cells/μL, despite the fact that the threshold CD4 count for ART initiation had been raised to 350 cells/μL in 2008 [32]. Another study from a large tertiary referral hospital in Beijing aimed to quantify the delay in time from the initial presentation to any healthcare settings to the ultimate HIV/AIDS diagnosis and found that the average length of diagnostic delay decreased from 91 days in 1997–2002 to 39 days in 2009–2012. However, the severity of disease at the time of diagnosis remained unchanged over the two time periods, as demonstrated by the fact that over 60% of newly diagnosed patients had CD4 counts below 50 cells/μL regardless of the time periods [33]. Furthermore, these patients presented with non-specific symptoms, and had sought care in various departments before ultimately being diagnosed with HIV/AIDS [34]. Provider-initiated HIV testing and counseling (PITC) is a critical strategy recommended by the WHO to expand access to HIV-related services. To accomplish this, it is important to enhance the awareness of HIV infection and its clinical diversity among healthcare providers across subspecialties. Concurrently, it is equally critical to enhance education and awareness among high-risk populations, especially in high prevalence areas.

#### Drug Resistance

The emergence of HIV drug resistance is a major problem that limits the effectiveness of ART. China has traditionally been recognized as a country with low prevalence of transmitted drug resistance (TDR), or primary drug resistance, compared with that observed in some industrialized countries, and rates have remained relatively stable over time. One study compared the prevalent ratio of drug-resistant variants before and after the introduction of NFATP in China, and found no significant difference in overall prevalence of resistance between 1991 and 2004 (7.1%) and 2005 and 2009 (6.7%), nor did the authors detect any differences within specific classes of antiretrovirals over time, including nucleoside reverse transcriptase inhibitors (NRTIs, 2.5%), non-nucleoside reverse transcriptase inhibitors (NNRTIs, 2.3%), and protease inhibitors (PIs, 2.7%) [35]. In 2015, a nationwide cross-sectional survey including 5627 newly diagnosed treatment-naïve patients was conducted, and the average rate of TDR mutations was 3.6%, with 1.1% resistant to PIs, 1.3% resistant to NRTIs, and 1.6% to NNRTIs [36]. However, uneven geographic distribution of resistance has been reported, and certain areas have shown exceptionally high rates of TDR. A retrospective cohort study in Shanghai from 2008 to 2015 reported high rates of TDR, with the highest drug resistance rate found among NNRTIs at 10.9% [37]. Similarly, a cross-sectional study in Zhejiang province showed a TDR rate of 11.1% among treatment-naïve HIV-infected individuals during 2014–2017 [38]. In contrast, studies in Beijing continue to show relatively low rates of TDR. The reasons behind the wide variability in observed rates of TDR remain unclear; however, these findings deserve attention and may influence recommendations regarding the most appropriate first-line regimen for treatment-naïve patients from different areas. The emergence of acquired drug resistance (ADR) is mainly related to poor patient adherence or to selection pressure on HIV quasi-species of the initial ART regimen [39]. ADR is the primary driver of treatment failure, and studies have found rates of ADR to any-class drugs reached as high as 50–60% among treatment-failure individuals in certain regions of China [37, 40]. Therefore, enhanced education and monitoring with regard to adherence, and adequate intervals of viral surveillance, especially in the first year of treatment, play an essential role in the control of ADR. Beyond this, broadening access to antiretroviral drugs that target different virus-host interaction sites will continue to constitute an important strategy for managing HIV drug resistance.

#### Novel Strategies for Unresolved Immune Activation

Despite the successes of modern ART, HIV eradication remains an elusive goal to researchers worldwide. Even with decades of suppressive treatment, residual levels of viral replication persist, associated with unresolved low levels of immune activation, generating a vicious circle that results in incomplete immune reconstitution and increased risk for non-AIDS-related comorbidities [41, 42]. This presents a huge challenge not only to China but also to the global community. Although great efforts have been made in China to better monitor and treat non-AIDS-related complications, work in this field has just started and much remains to be elucidated. Concurrently, as residual inflammation is likely to be a critical determinant contributing to HIV persistence, approaches to alleviate immune activation and reduce the viral reservoir have also been tested [43]. Of note, it is estimated that 20% of HIV-infected patients fail to achieve adequate immunologic recovery despite well-suppressed viral replication, a phenomenon in which immune activation plays a crucial role. Immunosuppressive agents including chloroquine have been tested in combination with ART to improve immune reconstitution in such cases; however, no sustained benefit was demonstrated [44]. *Tripterygium wilfordii Hook F* (TwHF) is a well-documented immune modulating agent approved by CFDA that has been widely used in China for treating autoimmune diseases such as rheumatoid arthritis, with documented efficacy for controlling inflammation [45–47]. Inspired by the findings from the rheumatology literature, in recent years our group has explored the hypothesis that TwHF may also reduce residual immune activation in HIV infection. In a pilot study, eighteen HIV-infected patients on ART who were virologically suppressed for over 12 months with suboptimal CD4 cell recovery (< 350 cells/mm^3^) were enrolled and received TwHF for 12 months. TwHF coadministration resulted in a mean elevation of 88 cells/μL in CD4 counts and significant reduction of T cell activation [48]. At the present time, a multicenter, randomized controlled trial (Clinicaltrials.gov ID: NCT04084444) is underway, to test the efficacy of triptolide, a compound from TwHF extract that possesses active immunosuppressive and anti-inflammatory properties [49], in improving immune reconstitution in HIV-infected individuals. Results of this study may provide an answer to the unresolved questions of persisting immune activation in HIV.

## Conclusion

During the past 20 years, China has made tremendous efforts to increase access to ART regimens through the gradual expansion of the NFATP, and has continually sought to evaluate and optimize these regimens for Chinese patients. Serial domestic multicenter studies have confirmed the efficacy and tolerability of available antiretrovirals in Chinese patients, elucidated differences in toxicity profile for certain agents, presented novel strategies to minimize the occurrence of known adverse events, and identified affordable regimens that can be utilized in low-resource settings when patients are not candidates for the first-line regimens. These successes have enabled a dramatic reduction in mortality from HIV/AIDS over the past two decades, and the number of patients on long-term ART is now over 800,000. Nevertheless, late diagnosis and linkage to care remain significant obstacles, and long-term non-AIDS comorbidities represent a new area of challenge in HIV care. This has prompted proposals of comprehensive “all-in-one” HIV care centers that would not only physically link diagnosis and treatment, but also engage a trained, multidisciplinary team of health professionals in HIV care. Finally, future priorities include continued close surveillance of patterns in drug resistance, particularly ADR, within China, and investigation of novel therapies to reduce HIV-related chronic immune activation.

## References

[1] Hong Y, Zhou D (1986). Clinical pathological conference: fever, cough and progressive dyspnea. Chin J Internal Med.

[2] National Health Commission of the People's Republic of China: Regular press conference: Progress in the prevention and treatment of AIDS in China. http://ncaidschinacdccn/xxgx/yqxx/201811/t20181123_197488.htm Acessed at Aug 23rd, 2019.

[3] Zhang FJ, Pan J, Yu L, Wen Y, Zhao Y (2005). Current progress of China’s free ART program. Cell Res.

[4] Zhang F, Haberer JE, Wang Y, Zhao Y, Ma Y, Zhao D, Yu L, Goosby EP (2007). The Chinese free antiretroviral treatment program: challenges and responses. Aids.

[5] Li T, Dai Y, Kuang J, Jiang J, Han Y, Qiu Z, Xie J, Zuo L, Li Y (2008). Three generic nevirapine-based antiretroviral treatments in Chinese HIV/AIDS patients: multicentric observation cohort. PLoS One.

[6] Zhang C, Wang W, Zhou M, Han Y, Xie J, Qiu Z, Guo F, Li Y, Wang H, Ghanem K (2013). The interaction of CD4 T-cell count and nevirapine hepatotoxicity in China: a change in national treatment guidelines may be warranted. J Acquir Immune Defic Syndr.

[7] AIDS Professional Group (2011). Society of Infectious Diseases Chinese Medical Association: guidelines for diagnosis and treatment of HIV/AIDS in China (2011). ZHONGHUA CHUAN RAN BING ZA ZHI.

[8] Li T, Guo F, Li Y, Zhang C, Han Y, Lye W, He Y, Lu H, Xie J, Huang A (2014). An antiretroviral regimen containing 6 months of stavudine followed by long-term zidovudine for first-line HIV therapy is optimal in resource-limited settings: a prospective, multicenter study in China. Chin Med J.

[9] ENCORE1 Study Group: Efficacy of 400 mg efavirenz versus standard 600 mg dose in HIV-infected, antiretroviral-naive adults (2014). (ENCORE1): a randomised, double-blind, placebo-controlled, non-inferiority trial. Lancet.

[10] Carey D, Puls R, Amin J, Losso M, Phanupak P, Foulkes S, Mohapi L, Crabtree-Ramirez B, Jessen H, Kumar S (2015). Efficacy and safety of efavirenz 400 mg daily versus 600 mg daily: 96-week data from the randomised, double-blind, placebo-controlled, non-inferiority ENCORE1 study. Lancet Infect Dis.

[11] Guo F, Cheng X, Hsieh E, Du X, Fu Q, Peng W, Li Y, Song X, Routy JP, Li T (2018). Prospective plasma efavirenz concentration assessment in Chinese HIV-infected adults enrolled in a large multicentre study. HIV Med.

[12] AIDS and Hepatitis C Professional Group (2018). Society of Infectious Diseases Chinese Medical Association, Chinese Center for Disease Control and Prevention: [Chinese guidelines for diagnosis and treatment of HIV/AIDS (2018)]. Zhonghua Nei Ke Za Zhi.

[13] Chinese Medical Association, and Chinese Center for Disease Control and Prevention: Guidelines for diagnosis and treatment of HIV/AIDS in China (2005). Chin Med J. 2006;119(19):1589–608. 17042972

[14] AIDS Professional Group (2015). Society of Infectious Diseases, Chinese Medical Association: third edition of the guidelines for diagnosis and treatment of HIV/AIDS (2015). Chin J Clin Infect Dis.

[15] Lundgren J, Babiker A, Gordin F, Emery S, Grund B, Sharma S, Avihingsanon A, Cooper D, Fatkenheuer G, Llibre J (2015). Initiation of antiretroviral therapy in early asymptomatic HIV infection. N Engl J Med.

[16] He X, Xing H, Ruan Y, Hong K, Cheng C, Hu Y, Xin R, Wei J, Feng Y, Hsi J (2012). A comprehensive mapping of HIV-1 genotypes in various risk groups and regions across China based on a nationwide molecular epidemiologic survey. PLoS One.

[17] Li Y, Han Y, Xie J, Gu L, Li W, Wang H, Lv W, Song X, Li Y, Routy J, Ishida T, Iwamoto A, Li T, CACT0810 group (2014). CRF01_AE subtype is associated with X4 tropism and fast HIV progression in Chinese patients infected through sexual transmission. Aids.

[18] Teeraananchai S, Kerr SJ, Amin J, Ruxrungtham K, Law MG (2017). Life expectancy of HIV-positive people after starting combination antiretroviral therapy: a meta-analysis. HIV Med.

[19] Freiberg MS, Chang CC, Kuller LH, Skanderson M, Lowy E, Kraemer KL, Butt AA, Bidwell Goetz M, Leaf D, Oursler KA, Rimland D, Rodriguez Barradas M, Brown S, Gibert C, McGinnis K, Crothers K, Sico J, Crane H, Warner A, Gottlieb S, Gottdiener J, Tracy RP, Budoff M, Watson C, Armah KA, Doebler D, Bryant K, Justice AC (2013). HIV infection and the risk of acute myocardial infarction. JAMA Intern Med.

[20] Luo L, Zeng Y, Li T, Lv W, Wang H, Guo F, Han Y, Xie J, Qiu Z, Li Y, Song X, Zhu T, Zhang X, Li L, Ye Y, He Y, Lu H, Huang A, Tang X, Wang H, Zhang T, Gao G, Lei J, Wu X, Sun Y, Bai J, Li K, China AIDS Clinical Trial 0810 Group (2014). Prospective echocardiographic assessment of cardiac structure and function in Chinese persons living with HIV. Clin Infect Dis.

[21] Rosenberg AZ, Naicker S, Winkler CA, Kopp JB (2015). HIV-associated nephropathies: epidemiology, pathology, mechanisms and treatment. Nat Rev Nephrol.

[22] Cao Y, Gong M, Han Y, Xie J, Li X, Zhang L, Li Y, Song X, Zhu T, Li T (2013). Prevalence and risk factors for chronic kidney disease among HIV-infected antiretroviral therapy-naive patients in mainland China: a multicenter cross-sectional study. Nephrology(Carlton).

[23] Brown TT, Qaqish RB (2006). Antiretroviral therapy and the prevalence of osteopenia and osteoporosis: a meta-analytic review. Aids.

[24] Ye Y, Zeng Y, Li X, Zhang S, Fang Q, Luo L, Qiu Z, Han Y, Li T (2010). HIV infection: an independent risk factor of peripheral arterial disease. J Acquir Immune Defic Syndr.

[25] Li H, Zhang F, Lu H, Cai W, Wu H, Sun Y, Zhao H, Zhang T, Cao WITL (2017). Expert consensus on management of HIV infection combined with chronic renal diseases. Chin J AIDS&STD.

[26] AIDS Professional Group (2017). Society of Infectious Diseases Chinese Medical Association, Society of Tropical Diseases and Parasitology of Chinese Medical Association: expert consensus on diagnosis and treatment of HIV infection combined with mycobacterium tuberculosis infection. Chin J Clin Infect Dis.

[27] Society of Tropical Diseases and Parasitology of Chinese Medical Association (2019). Expert consensus on diagnosis and treatment of HIV infection combined with nontuberculosis mycobacterium infection. Chin J Infect Dis.

[28] Zhang F, Zhu H, Wu Y, Dou Z, Zhang Y, Kleinman N, Bulterys M, Wu Z, Ma Y, Zhao D, Liu X, Fang H, Liu J, Cai WP, Shang H (2014). HIV, hepatitis B virus, and hepatitis C virus co-infection in patients in the China National Free Antiretroviral Treatment Program, 2010-12: a retrospective observational cohort study. Lancet Infect Dis.

[29] Xie J, Han Y, Qiu Z, Li Y, Li Y, Song X, Wang H, Thio CL, Li T (2016). Prevalence of hepatitis B and C viruses in HIV-positive patients in China: a cross-sectional study. J Int AIDS Soc.

[30] Wang H, Li Y, Zhang C, Han Y, Zhang X, Zhu T, Li T (2012). Immunological and virological responses to cART in HIV/HBV co-infected patients from a multicenter cohort. Aids.

[31] Li Y, Xie J, Han Y, Wang H, Zhu T, Wang N, Lv W, Guo F, Qiu Z, Li Y (2016). Lamivudine Monotherapy-Based cART Is Efficacious for HBV Treatment in HIV/HBV Coinfection When Baseline HBV DNA <20,000 IU/mL. J Acquir Immune Defic Syndr.

[32] Tang H, Mao Y, Tang W, Han J, Xu J, Li J (2018). "Late for testing, early for antiretroviral therapy, less likely to die": results from a large HIV cohort study in China, 2006-2014. BMC Infect Dis.

[33] Xie J, Hsieh E, Sun M, Wang H, Lv W, Fan H, Li T (2017). Delays in HIV diagnosis and associated factors among patients presenting with advanced disease at a tertiary care hospital in Beijing, China. PLoS One.

[34] Cao W, Song X, Li Y, Qiu Z, Xie J, Han Y, Lyu W, Wang H, Fan H (2014). Zhou B et al: [Clinical characteristics of 297 newly diagnosed Chinese HIV/AIDS patients]. Zhonghua Nei Ke Za Zhi.

[35] Li HP, Chang SA, Han Y, Zhuang DM, Li L, Liu YJ, Liu SY, Bao ZY, Zhang WF, Song HB (2016). The prevalence of drug resistance among treatment-naive HIV-1-infected individuals in China during pre- and post-2004. BMC Infect Dis.

[36] Zhao S, Feng Y, Hu J, Li Y, Zuo Z, Yan J, Zhang J, Cao P, Xu W, Li F, Li Y, Liao L, Ruan Y, Shao Y, Xing H (2018). Prevalence of transmitted HIV drug resistance in antiretroviral treatment naive newly diagnosed individuals in China. Sci Rep.

[37] Zhang FD, Liu L, Sun MY, Sun JJ, Lu HZ (2017). An analysis of drug resistance among people living with HIV/AIDS in Shanghai, China. PLoS One.

[38] Xu Y, Peng X, Peng X, Ji S, Chen B, Wang L, Lu X, Xie T, Sun T, Wang H, Wu N (2018). Characterization of HIV-1 subtypes and transmitted drug resistance among treatment-naive HIV-infected individuals in Zhejiang, China, 2014-2017. Arch Virol.

[39] Ma Y, Zhao D, Yu L, Bulterys M, Robinson ML, Zhao Y, Dou Z, Chiliade P, Wei X, Zhang F (2010). Predictors of virologic failure in HIV-1-infected adults receiving first-line antiretroviral therapy in 8 provinces in China. Clin Infect Dis.

[40] Wu J, Norris J, Liu HX, Li Z, Su YY, Zhu L, Wang N (2014). The prevalence of HIV drug resistance among treatment-failure individuals and treatment-naive individuals in China: a meta-analysis. Biomed Environ Sci.

[41] Klatt NR, Chomont N, Douek DC, Deeks SG (2013). Immune activation and HIV persistence: implications for curative approaches to HIV infection. Immunol Rev.

[42] Li T, Wu N, Dai Y, Qiu Z, Han Y, Xie J, Zhu T, Li Y (2011). Reduced thymic output is a major mechanism of immune reconstitution failure in HIV-infected patients after long-term antiretroviral therapy. Clin Infect Dis.

[43] Massanella M, Fromentin R, Chomont N (2016). Residual inflammation and viral reservoirs: alliance against an HIV cure. Curr Opin HIV AIDS.

[44] Routy JP, Angel J, Patel M, Kanagaratham C, Radzioch D, Kema I, Gilmore N, Ancuta P, Singer J, Jenabian MA (2015). Assessment of chloroquine as a modulator of immune activation to improve CD4 recovery in immune nonresponding HIV-infected patients receiving antiretroviral therapy. HIV Med.

[45] Tao X, Younger J, Fan FZ, Wang B, Lipsky PE (2002). Benefit of an extract of Tripterygium Wilfordii Hook F in patients with rheumatoid arthritis: a double-blind, placebo-controlled study. Arthritis Rheum.

[46] Lv QW, Zhang W, Shi Q, Zheng WJ, Li X, Chen H, Wu QJ, Jiang WL, Li HB, Gong L (2015). Comparison of Tripterygium wilfordii Hook F with methotrexate in the treatment of active rheumatoid arthritis (TRIFRA): a randomised, controlled clinical trial. Ann Rheum Dis.

[47] Goldbach-Mansky R, Wilson M, Fleischmann R, Olsen N, Silverfield J, Kempf P, Kivitz A, Sherrer Y, Pucino F, Csako G (2009). Comparison of Tripterygium wilfordii Hook F versus sulfasalazine in the treatment of rheumatoid arthritis: a randomized trial. Ann Intern Med.

[48] Li T, Xie J, Li Y, Routy JP, Li Y, Han Y, Qiu Z, Lv W, Song X, Sun M, Zhang X, Wang F, Jiang H (2015). Tripterygium wilfordii Hook F extract in cART-treated HIV patients with poor immune response: a pilot study to assess its immunomodulatory effects and safety. HIV Clin Trials.

[49] Kusunoki N, Yamazaki R, Kitasato H, Beppu M, Aoki H, Kawai S (2004). Triptolide, an active compound identified in a traditional Chinese herb, induces apoptosis of rheumatoid synovial fibroblasts. BMC Pharmacol.

